# The Hunger Games: *Aggregatibacter actinomycetemcomitans* Exploits Human Neutrophils As an Epinephrine Source for Survival

**DOI:** 10.3389/fimmu.2021.707096

**Published:** 2021-08-12

**Authors:** Hazel Ozuna, Silvia M. Uriarte, Donald R. Demuth

**Affiliations:** ^1^Department of Microbiology and Immunology, School of Medicine, University of Louisville, Louisville, KY, United States; ^2^Department of Oral Immunology and Infectious Diseases, School of Dentistry, University of Louisville, Louisville, KY, United States

**Keywords:** *Aggregatibacter actinomycetemcomitans*, human neutrophils, catecholamines, epinephrine, granule exocytosis, QseBC system, periodontitis

## Abstract

*Aggregatibacter actinomycetemcomitans* is a gram-negative facultative anaerobe and an opportunistic oral pathogen, strongly associated with periodontitis and other inflammatory diseases. Periodontitis is a chronic inflammation of the periodontium resulting from the inflammatory response of the host towards the dysbiotic microbial community present at the gingival crevice. Previously, our group identified catecholamines and iron as the signals that activate the QseBC two-component system in *A. actinomycetemcomitans*, necessary for the organism to acquire iron as a nutrient to survive in the anaerobic environment. However, the source of catecholamines has not been identified. It has been reported that mouse neutrophils can release catecholamines. In periodontitis, large infiltration of neutrophils is found at the subgingival pocket; hence, we wanted to test the hypothesis that *A. actinomycetemcomitans* exploits human neutrophils as a source for catecholamines. In the present study, we showed that human neutrophils synthesize, store, and release epinephrine, one of the three main types of catecholamines. Human neutrophil challenge with *A. actinomycetemcomitans* induced exocytosis of neutrophil granule subtypes: secretory vesicles, specific granules, gelatinase granules, and azurophilic granules. In addition, by selectively inhibiting granule exocytosis, we present the first evidence that epinephrine is stored in azurophilic granules. Using QseC mutants, we showed that the periplasmic domain of the QseC sensor kinase is required for the interaction between *A. actinomycetemcomitans* and epinephrine. Finally, epinephrine-containing supernatants collected from human neutrophils promoted *A. actinomycetemcomitans* growth and induced the expression of the *qseBC* operon under anaerobic conditions. Based on our findings, we propose that *A. actinomycetemcomitans* promotes azurophilic granule exocytosis by neutrophils as an epinephrine source to promote bacterial survival.

**Graphical Abstract:**
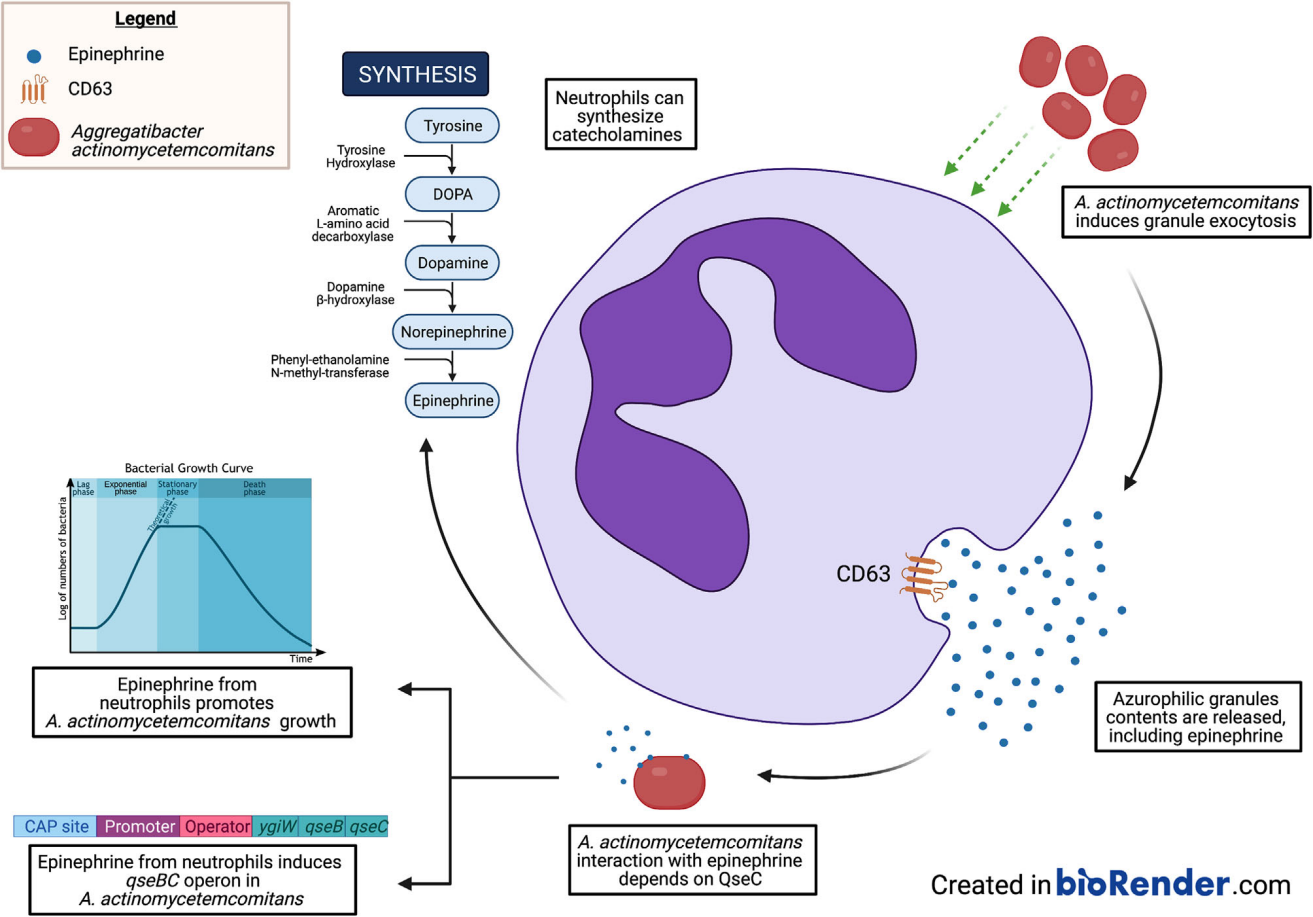


## Introduction

In the oral cavity, when host microbe homeostasis is broken, bacterial communities accumulate at the sub-gingival pocket and form biofilms that lead to oral diseases such as periodontitis (1). Periodontitis consists of a chronic inflammation of the periodontium caused by the inflammatory response of the host to plaque biofilm. The disease is characterized by the progressive deepening of the sulcus and loss of attachment between the bone and gingival tissue leading to bone loss. Recurring inflammation of the periodontium has been associated with the initiation, exacerbation, and pathogenesis of a number of other inflammatory diseases (2). *Aggregatibacter actinomycetemcomitans* (*Aa*) is a non-motile gram-negative facultative anaerobe of the *Pasteurellaceae* family (3–5). *A. actinomycetemcomitans* has been strongly associated with periodontitis and other diseases such as rheumatoid arthritis, cardiovascular diseases, atherosclerosis, urinary tract infections, and brain abscesses (6–10). *A. actinomycetemcomitans* contributes to tissue inflammation, destruction, and bone resorption by expressing a number of virulence factors such as cytolethal disentin toxin, leukotoxin A of the RTX family of bacterial toxins, and collagenase (11–14).

In the subgingival pocket, *Aa* must compete for nutrients, such as iron, in order to survive. As part of the host immune response, iron is kept unavailable to bacteria by being sequestered by catecholamines (i.e., epinephrine, norepinephrine, and dopamine) or leukocyte-produced molecules like lactoferrin and transferrin (15, 16). However, some bacteria have evolved to subvert this mechanism by producing iron scavenging molecules, known as siderophores (17–19) or using host-derived catecholamines (15). Catecholamines have been implicated as causative or contributory agents of periodontitis (20–22). Interestingly, *Aa* does not produce siderophores (23), but expresses the QseBC (quorum sensing in *Escherichia coli*) two-component system (TCS) that is activated in the presence of both catecholamines and iron (6). The *qseBC* operon encodes the genes for *qseC*, a sensor molecule, and *qseB*, the response regulator (24). Novak et al. (25) demonstrated the requirement of QseC for *Aa* biofilm growth and virulence. Furthermore, Weigel et al. (6) subsequently showed that *Aa* growth in a chemically defined media (CDM) supplemented with both epinephrine or norepinephrine and ferrous (Fe^2+^) or ferric (Fe^3+^) iron increased *qseBC* promoter activity and bacterial growth, indicating that both catecholamines and iron are required for the activation of QseBC. Additionally, microarray analysis showed that activation of QseBC induced the expression of genes associated with anaerobic metabolism and respiration and reduced expression of genes involved in iron uptake and transport. However, the source of catecholamines remained to be determined. In periodontitis, neutrophils are found in large numbers at the gingival crevice (26). Murine neutrophils have been shown to release epinephrine or norepinephrine when stimulated with LPS (27). Furthermore, increased levels of the enzymes (tyrosine hydroxylase and dopamine β-hydroxylase) required for the synthesis of catecholamines and their inactivation (catecholamine-O-methyltransferase and monoamine oxidase) enzymes have been observed in murine phagocytes and human lymphocytes (27, 28). Nonetheless, the presence of catecholamines in the host environment has also been shown to promote bacterial iron uptake and growth in media limited conditions (15, 21, 22). Host-derived catecholamines have a higher affinity to iron than antimicrobials like lactoferrin and transferrin (15, 29), serving as excellent pseudo-siderophores (30) to bacteria. Therefore, we proposed that human neutrophils serve as a catecholamine source for *Aa* to sequester iron, leading to QseBC activation and bacterial growth.

In this study, we demonstrate that human neutrophils have significant levels of tyrosine hydroxylase, monoamine oxidase A, and catecholamine-O-methyltransferase, validating that cells participate in catecholamine metabolism. In addition, we present evidence that human neutrophils store epinephrine in the azurophilic granules and that interaction with *Aa* induces exocytosis of azurophilic granules and release of epinephrine. Furthermore, we show that treatment with latrunculin A followed by fMLF stimulation, which is known to induce mobilization and content release of azurophilic granules, induces epinephrine release in human neutrophils. The interaction of released epinephrine with *Aa* activates QseBC and induces *Aa* growth under anaerobic conditions. These results suggest that neutrophils may serve as an epinephrine source for *Aa*, to facilitate growth and prime anaerobic metabolism in the subgingival pocket. The findings presented in this article help shed light into the crosstalk that exits between bacteria and the host endocrine system by providing an example of the role that stress hormones play in periodontal disease and potentially other chronic inflammatory diseases.

## Methods

### Human Neutrophil Isolation and Purification

Recruitment of human donors, blood draws, and use of required materials were done in agreement with guidelines approved by the Institutional Review Board of the University of Louisville. Human neutrophils were isolated from whole blood of healthy donors using plasma-Percoll gradients as previously described (31). When necessary, neutrophils were further purified to obtain >99% pure population. Purification was carried by negative magnetic selection using the Easy Sep Human neutrophil isolation kit (Stemcell Technologies, Vancouver, BC, Canada). Cell purity was assessed by simultaneously staining with FITC-conjugated anti-CD66b (clone G10F5; BioLegend, San Diego, CA, USA) and APC-conjugated anti-CD16 (clone CB16; eBioscience, San Diego, CA, USA) antibodies and determining the percentage of CD66b**^+^**CD16**^+^** cells using a BD Celesta flow cytometer (BD Biosciences, San Jose, CA, USA) and FlowJo for analysis (FlowJo, LLC, Ashland, OR, USA). Both pure (>90%–95%) and highly pure (>99%) neutrophils were cultured in RPMI-1640 medium without phenol red (Sigma-Aldrich, St. Louis, MO, USA) supplemented with 2 mM L-glutamine (Sigma-Aldrich, St. Louis, MO, USA) and 5% human serum (Atlanta Biologicals, Flowery Branch, GA, USA).

### Bacteria Strains and Media

In this study, we make use of *A. actinomycetemcomitans* 652 serotype c strain, a low leukotoxic, afimbriated, smooth colony-morphotype variant. Considered an opportunistic pathogen, this strain has been isolated from healthy and in large numbers in periodontitis-positive individuals (32, 33). At sublytic concentrations of leukotoxin A, there is an increase in calcium levels and neutrophil activation leading to oxidative burst and degranulation (34)*. A. actinomycetemcomitans* strains (**Supplementary Table 1**) were propagated in brain–heart infusion (BHI, Difco, BD Biosciences, Franklin Lakes, NJ, USA) broth supplemented with bacitracin (50 µg/ml) and vancomycin (50 µg/ml), unless indicated otherwise. *A. actinomycetemcomitans* was grown at 37°C under microaerophilic conditions in a closed tube. The *Aa* mutant strains (all in *Aa* 652 background, see **Supplementary Table 1**) used for epinephrine interaction experiments were previously constructed by Juarez-Rodríguez et al. (24): *Aa ΔqseC* (non-polar *qseC* gene deletion mutant, spectinomycin 50 µg/ml), *Aa qseCΔp* (QseC sensor protein with an in-frame deletion of the periplasmic sensor domain, spectinomycin 50 µg/ml), and *Aa ΔqseC-comp* (non-polar *qseC* gene deletion mutant complemented with a single genomic copy of the *qseC* gene, spectinomycin 50 µg/ml). The *Aa* strain expressing the plasmid pDJR29, which contains the *lacZ* gene controlled by the qseBC promoter (35), was used in β-galactosidase assays and was grown under anaerobic conditions in CDM (**Supplementary Table 2**) (36), supplemented with kanamycin (25 µg/ml). All reagents used in anaerobic experiments were oxygen-depleted.

*Filifactor alocis (F. alocis)* ATCC 38596 was cultured in BHI broth supplemented with 5 mg/ml yeast extract, L-cysteine (0.05%), and arginine (0.05%) for 7 days anaerobically at 37°C as previously described (36–38).

### *A. actinomycetemcomitans* Challenge, Epinephrine Detection, and Neutrophil Viability

Human neutrophils (3 × 10^6^ cells/ml) were challenged in suspension with *Aa* at a multiplicity of infection (MOI) (39) of 50 at 37°C in a shaking water bath for 2 h, 4 h, 8 h, and 24 h, unless indicated otherwise. As a positive control for epinephrine release, neutrophils were treated with latrunculin A (1 μM, Sigma-Aldrich, St. Louis, MO, USA) for 30 min, followed by stimulation with N-formylmethionyl-leucyl-phenylalanine (fMLF, 300 nM, Sigma-Aldrich, St. Louis, MO, USA) for 5 min at 37°C in a shaking water bath. At the end of incubation, samples were centrifuged at 6,000 × *g* for 30 s and supernatants were collected and supplemented with 100× Halt Protease and Phosphatase inhibitor single-use cocktail (1:10 dilution; Thermo Fisher Scientific, Waltham, MA, USA). The pelleted cells were lysed with ice-cold 1× lysis buffer (10 µl per 1 × 10^6^ of cells; [20 mM Tris-HCl [pH 7.5], 150 mM NaCl, 1% [vol/vol] Triton X-100, 0.5% [vol/vol] Nonidet P-40, 20 mM NaF, 20 mM NaVO_3_, 1 mM EDTA, 1 mM EGTA, 5 mM phenylmethylsulfonyl fluoride [PMSF], 2 mM diisopropylfluorophosphate [DFP], 21 μg/ml aprotinin, and 5 μg/ml leupeptin]). Lysates were centrifuged and cell lysate was supplemented with 100× Halt Protease and Phosphatase inhibitor single-use cocktail (1:10 dilution; Thermo Fisher Scientific, Waltham, MA, USA). Epinephrine was measured in supernatants and cell lysate with a commercially available Adrenaline/Epinephrine ELISA kit (Cat. No. E4359, BioVision Inc., Milpitas, CA, USA).

Neutrophil viability when challenged with *Aa* was determined by Trypan Blue exclusion and cytospin microscopy imaging. For Trypan Blue exclusion, neutrophils were diluted 1:20 in Trypan blue at 0 h, 4 h, 8 h, and 24 h, and live cells were counted using a hemacytometer. For cytospin images, cells were centrifuged at 6,000 × *g* for 30 s and washed twice with RPMI-1640 (no phenol red, Sigma-Aldrich, St. Louis, MO, USA) to remove bacteria. Neutrophils were resuspended at 1 × 10^5^ cells in 200 µl of RPMI-1640 and 5 µl of human serum was added (Atlanta Biologicals, Flowery Branch, GA, USA). The cell suspension was loaded into the funnel chamber that is assembled on the cytocentrifuge clip, with slide and filter. Cytocentrifuge clip was centrifuged for 5 min at 800 rpm (Shandon Cytospin 3, Thermo Fisher Scientific, Waltham, MA, USA). The microscope slide was removed from the cytocentrifuge clip and fixed and stained using the Hema 3 Protocol staining kit (Thermo Fisher Scientific, Waltham, MA, USA).

### Catecholamine Metabolism Enzyme-Linked Immunosorbent Assay (ELISA)

Cell lysates collected from neutrophils challenged with *Aa* at 2 h, 4 h, 8 h, and 24 h were tested for the presence of enzymes involved in catecholamine metabolism. The levels of tyrosine hydroxylase (Cat. No. NBP3-06920, Novus Biologicals, CO, USA), dopamine β-hydroxylase (Cat. No. NBP2-67945, Novus Biologicals, CO, USA), catechol-o-methyltransferase (Cat. No. OKBB00966, Aviva Systems Biology Corp., San Diego, CA, USA), and monoamine oxidase-A (Cat. No. OKEH02825, Aviva Systems Biology Corp., San Diego, CA, USA) were measured following the indicated protocols supplied by the kit manufacturer.

### Neutrophil Granule Exocytosis and Exocytosis Inhibition

Neutrophils (4 × 10**^6^** cells/ml) were challenged with fMLF (300 nM, 5 min), latrunculin A (1 μM, 30 min) + fMLF (300 nM, 5 min), or *Aa* at various time points from 5 min to 24 h at 37°C in a shaking water bath. The exocytosis of secretory vesicles, specific granules, and azurophilic granules was determined by measuring the plasma membrane increase of granule markers using fluorescein isothiocyanate (FITC)-conjugated anti-CD63 (for azurophilic granules, Ancell 215-040, Stillwater, MN, USA), FITC-conjugated anti-CD66b (for specific granules, Biolegend 305104, San Diego, CA, USA), and phycoerythrin (PE)-conjugated anti-CD35 (for secretory vesicles, Biolegend 333406, San Diego, CA, USA) by a FACSCalibur flow cytometer as previously described (31, 40). Following antibody incubation, cells were washed with 0.5% sodium azide (S2002, Sigma, St. Louis, MO, USA) in FTA buffer (211248 BD, Franklin Lakes, NJ, USA) and fixed with 1% paraformaldehyde (PX0055-3, EMD, Darmstadt, Germany). Gelatinase granule exocytosis was determined by measuring the release of matrix metallopeptidase 9 (MMP-9) by ELISA (Cat. No. ab100610, Abcam, Cambridge, MA, USA).

TAT-SNAP23 and TAT-Syntaxin 4 were used to inhibit neutrophil granule exocytosis as previously described by Uriarte et al. (31) and McLeish et al. (40). Briefly, neutrophils (4 × 10**^6^** cells/ml) were pretreated with TAT-Syntaxin 4 (0.8 μg/ml), TAT-SNAP23 (0.8 μg/ml), or TAT-control (1 μg/ml) for 15 min, followed by challenge with *Aa* for 15 min, 2 h, 4 h, and 8 h at 37°C in a shaking water bath. Granule exocytosis was measured by increased plasma membrane expression of CD35 (secretory vesicles), CD66b (specific granules), and CD63 (azurophilic granules) by flow cytometry as described in a previous paragraph. In addition, cell supernatants were collected to measure release of secretory vesicle content by ELISA for albumin (Cat. No. EHALB, Invitrogen, Thermo Fisher Scientific, Waltham, MA, USA), gelatinase content by ELISA for MMP-9, specific granule content by ELISA for lactoferrin (Cat. No. ab108882, Abcam, Cambridge, MA, USA), and azurophilic granule content by ELISA for myeloperoxidase (Cat. No. ab119605, Abcam, Cambridge, MA, USA).

### Recombinant Epinephrine Interaction With *A. actinomycetemcomitans*

Epinephrine interaction with *Aa*: Increasing colony-forming units (CFUs) of *Aa* (0.0–4.0 × 10^8^ CFUs) were incubated with 250 pg/ml of recombinant epinephrine (BioVision Inc., Milpitas, CA, USA) for 15 min at 37°C in a shaking water bath. As a control, wells with epinephrine alone were run in parallel. Samples were centrifuged at 6,000 × *g* for 5 min, and supernatants were collected. Immediately after, epinephrine was measured from supernatants with a commercially available Adrenaline/Epinephrine ELISA kit (Cat. No. E4359, BioVision Inc., Milpitas, CA, USA). For analysis, interpolated values were obtained from recombinant epinephrine standard curve, following manufacturer instructions. The amount of epinephrine associated with *Aa* was determined by subtracting the interpolated values of bacteria-containing wells from the values obtained for control wells.

Epinephrine interaction with *Aa qseC* mutant strains: Mutant strains of *Aa* (*ΔqseC, qseC/Δp*, and *ΔqseC-comp*) were incubated at 3.0 × 10^7^ CFUs with increasing concentrations (0–500 pg/ml) of recombinant epinephrine (BioVision Inc., Milpitas, CA, USA) for 15 min at 37°C in a shaking water bath. Wells containing increasing concentrations of recombinant epinephrine serve as control. Afterwards, samples were centrifuged as above, and supernatants were collected. Immediately after, epinephrine was measured and analyzed as described above. The amount of epinephrine associated with *Aa* was determined by subtracting the interpolated values of *Aa*-containing wells from the values of epinephrine control.

### *A. actinomycetemcomitans* Growth Kinetics

*A. actinomycetemcomitans* wild type was inoculated into BHI broth supplemented with bacitracin (50 µg/ml) and vancomycin (50 µg/ml) and grown under microaerophilic conditions at 37°C to an optical density at 600 nm (OD**_600_**) of 0.3–0.4. Cells were subcultured in fresh BHI broth at a 1:30 dilution and grown as described above to an OD_600_ of 0.5–0.6. Cells were washed with CDM (**Supplementary Table 2**) and inoculated into CDM supplemented as above at a 1:30 dilution. Cultures were supplemented individually or with a combination of 100 µM FeCl**_2_**, 50 µM epinephrine, supernatant, or cell lysate collected from neutrophils stimulated with latrunculin A and fMLF (final epinephrine concentration of 30 pg/ml). The OD_600_ was measured at various time points using a Bio-Rad SmartSpec Plus UV-vis spectrophotometer (Bio-Rad, Hercules, CA, USA).

### β-galactosidase Assay

Quantitative evaluation of β-galactosidase activity was determined using permeabilized cells incubated with o-nitrophenyl-b-D-galactopyranoside (ONPG) substrate (Sigma, St Louis, MO, USA) as previously described (41). Briefly, a primary culture of *Aa* (pDJR29) was grown at 37°C under microaerophilic conditions in a closed tube overnight in BHI broth. The culture was subcultured at a 1:30 dilution in BHI broth and grown as described in the previous paragraph for 24 h. Subsequently, the secondary overnight culture (OD_600_ of 0.3–0.4) was diluted 1:30 into CDM and grown in an anaerobic chamber for 24 h at 37°C. An aliquot of 0.1 ml was then used to determine the OD_600_ of the culture at 24 h and triplicate aliquots of 0.1 ml were used to measure β-galactosidase activity.

### Statistical Analysis

Unless otherwise noted, statistical experimental conditions and time points were analyzed by a one-way ANOVA, followed by post-hoc Tukey’s multi-comparison test using GraphPad Prism Software (GraphPad PRISM v9, San Diego, CA, USA). Differences were considered significant at *p* < 0.05.

## Results

### *A. actinomycetemcomitans* Challenge Induces Epinephrine Release and Promotes *De Novo* Catecholamine Synthesis in Human Neutrophils

**Figure 1:**
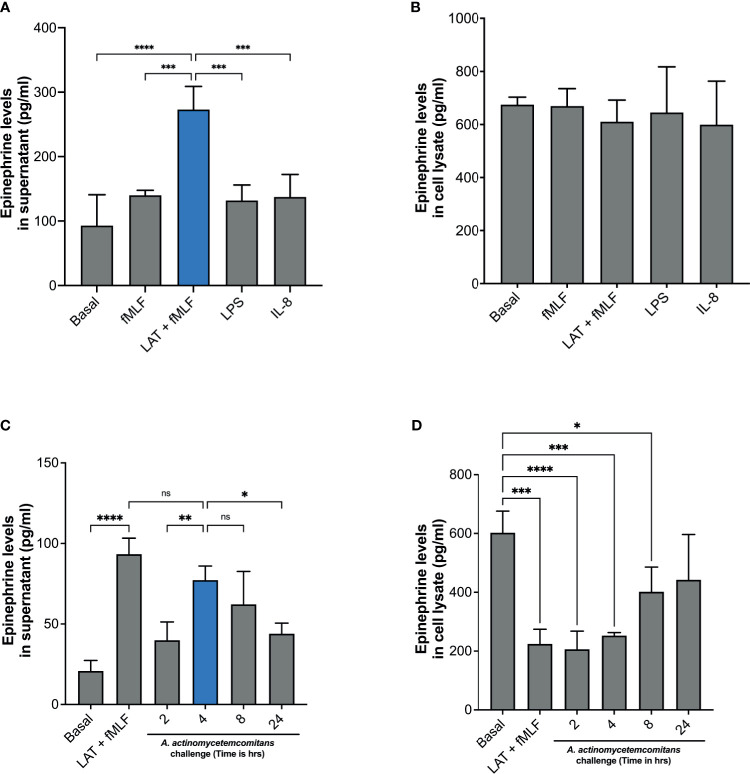
Human neutrophils release epinephrine upon *A*. *actinomycetemcomitans* challenge. Human neutrophils (5 × 10^6^ cells/ml) were treated with fMLF, Latrunculin A (LAT) + fMLF, LPS, or IL-8 to induce epinephrine release. Epinephrine content was determined in neutrophil supernatant **(A)** and cell lysate **(B)** by ELISA. Human neutrophils (3 × 10^6^ cells/ml) were challenged with *A*. *actinomycetemcomitans* (*Aa*; MOI 50) for 2 h, 4 h, 8 h, and 24 h At the end of each time point, supernatant **(C)** and cell lysates **(D)** were collected and epinephrine content was measured by ELISA. Epinephrine concentration is plotted as the mean ± SD of four independent experiments. Statistical differences among experimental conditions and time points were analyzed by one-way ANOVA **(A, B)** or repeated measures one-way ANOVA **(C, D)**, followed by Tukey’s post-test. ns, not significant, *p < 0.05, **p < 0.01, ***p < 0.001, ****p < 0.0001.

It has been previously shown that mouse neutrophils stimulated with LPS can release catecholamines (Flier 2007). However, there is as of yet no clear evidence of human neutrophils’ ability to release catecholamines. To determine if human neutrophils release epinephrine, cells were stimulated with fMLF, latrunculin A + fMLF, LPS, or IL-8. Supernatants and cell lysates were collected, and epinephrine levels were determined by ELISA. Unlike what was described in murine neutrophils (27), LPS stimulation did not induce epinephrine release by human neutrophils (**Figure 1A**). Similarly, stimulation of neutrophils with fMLF or IL-8 failed to induce epinephrine release (**Figure 1A**). Interestingly, treatment with latrunculin A followed by fMLF induced significant release of epinephrine compared to fMLF alone and basal (**Figure 1A**). Significant levels of epinephrine were detected in the cell lysates but there was no significant difference between experimental conditions (**Figure 1B**). Afterward, we examined the ability of *Aa* to induce epinephrine release by human neutrophils. Cells were challenged with *Aa* at a MOI of 50 in suspension for 2 h, 4 h, 8 h, and 24 h. Epinephrine levels were measured from supernatants and cell lysates by ELISA. **Figure 1C** shows that *Aa* challenge induced significant release of epinephrine compared to basal, with maximum levels reached at 4 h. Epinephrine levels in cell lysates of human neutrophils treated with latrunculin A + fMLF had significantly reduced levels of epinephrine compared to basal (**Figure 1D**); this correlates the significant increase release of epinephrine observed in supernatants (**Figure 1C**). Similarly, low epinephrine levels in neutrophil cell lysates were detected after challenged with *Aa* for 2 h and 4 h, which could be related to the release observed in the supernatants (**Figures 1C, D**). Interestingly, epinephrine release at 2 h was similar to basal (**Figure 1C**). A potential explanation for this is that the released epinephrine could be interacting in some way with *Aa*. We repeated this experiment with highly purified (>99%) human neutrophils (**Supplementary Figure 1**) and obtained similar results.

To confirm that the release of epinephrine is not caused by a cytotoxic effect of *Aa* on neutrophils, we evaluated the morphology of human neutrophils with cytospin imaging and viability with trypan blue exclusion. Human neutrophil morphology was not altered with *Aa* challenge (**Supplementary Figures 2A, B**) and challenge with *Aa* extended the lifespan of neutrophils (**Supplementary Figure 2C**) up to 24 h. Taken together, our results demonstrate that *Aa* stimulates epinephrine release in human neutrophils, depleting storage reservoirs. In addition, the difference in released (**Figure 1C**) versus stored epinephrine at 2 h (**Figure 1D**) suggests that epinephrine may be interacting with *Aa*. The increase of epinephrine levels in cell lysates at later time points of 8 h and 24 h (**Figure 1D**) suggests that human neutrophils are capable of *de novo* catecholamine synthesis.

**Figure 2:**
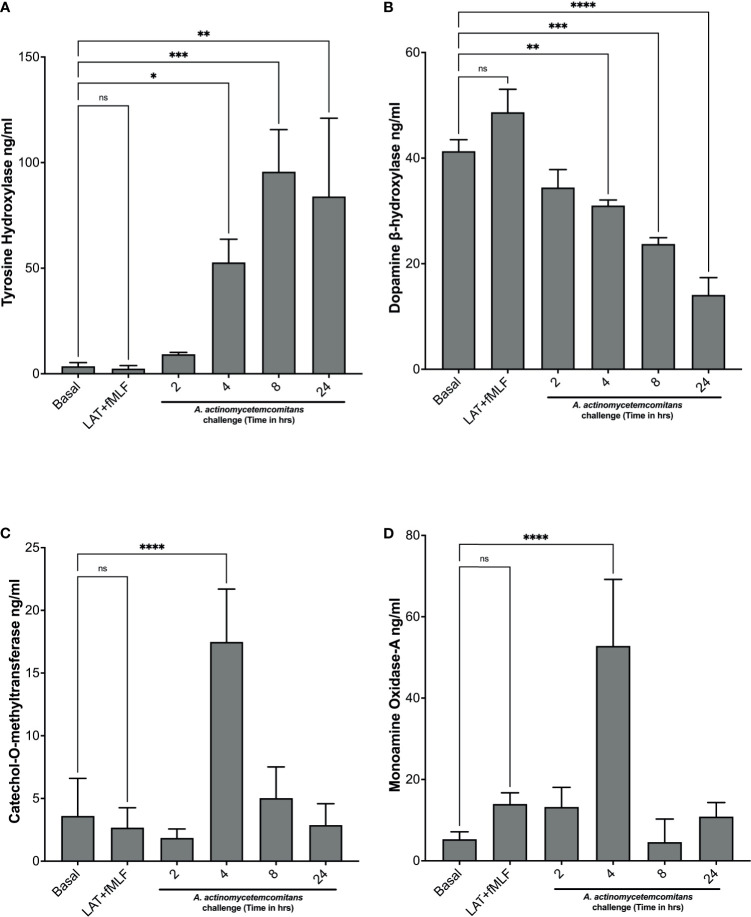
*A. actinomycetemcomitans* promotes increased levels of enzymes involved in catecholamine metabolism in human neutrophils. Human neutrophils were challenged with *A*. *actinomycetemcomitans* (MOI 50) for 2 h, 4 h, 8 h and 24 h; cell lysates were collected, and the levels of tyrosine hydroxylase **(A)**, dopamine β-hydroxylase **(B)**, catechol-O-methyltransferase **(C)**, and monoamine oxidase A **(D)** were measured by ELISA. Enzyme levels are plotted as the mean ± SD of three independent experiments. Statistical differences among experimental conditions and time points were analyzed by repeated measures one-way ANOVA, followed by Tukey’s post-test. ns, not significant, *p < 0.05, **p < 0.01, ***p < 0.001, ****p < 0.0001.

Next, we examined the levels of enzymes involved in catecholamine metabolism. Levels of enzymes involved in catecholamine synthesis—tyrosine hydroxylase and dopamine β-hydroxylase—and levels of enzymes involved in catecholamine inactivation—catechol-o-methyltransferase and monoamine oxidase-A—were measured in cell lysates of human neutrophils exposed to *Aa* at 2 h, 4 h, 8 h, and 24 h by ELISA. In the absence of *Aa*, tyrosine hydroxylase levels are significantly low, but after *Aa* challenge, levels increase significantly with time compared to basal (**Figure 2A**). In contrast, dopamine β-hydroxylase levels decrease when *Aa* is present (**Figure 2B**). The levels of catechol-o-methyltransferase (**Figure 2C**) and monoamine oxidase-A (**Figure 2D**) remained comparable to basal, only peaking at 4 h. Catecholamine synthesis enzyme (tyrosine hydroxylase levels) increase at 8 h and 24 h (**Figure 2A**), whereas inactivation enzymes (**Figures 2C, D**) decrease at the same time points. These results indicate that *Aa* challenge induces *de novo* catecholamine synthesis in human neutrophils.

In the infected subgingival pocket, *Aa* functions in consortium with other bacteria in bacterial communities known as biofilms and Fine et al. showed that *A. actinomycetemcomitans* positive subjects with bone loss had high levels of the oral pathogen *F. alocis* (42). In addition, Wang et al. demonstrated that *F. alocis* accumulation in the oral biofilm was stimulated by the presence of specific strains of *Aa* (43). To examine if the induction of epinephrine release is specific to *Aa*, we performed a time course experiment where we investigated the ability of *F. alocis* to induce epinephrine release in human neutrophils. Human neutrophils were challenged with *F. alocis* at a MOI of 10 at 1 h, 2 h, and 4 h incubation and epinephrine detection was performed as described above. *F. alocis* failed to induce significant release of epinephrine (**Supplementary Figure 3A**). Consistent with this, no significant difference in epinephrine content was detected in cell lysates among experimental conditions (**Supplementary Figure 3B**). In addition, human neutrophils were challenged with LPS up to 24 h and no significant epinephrine release or difference in epinephrine content was observed (**Supplementary Figures 3C, D**).

### *A. actinomycetemcomitans* Access Epinephrine by Inducing Exocytosis of Human Neutrophil Granules

**Figure 3:**
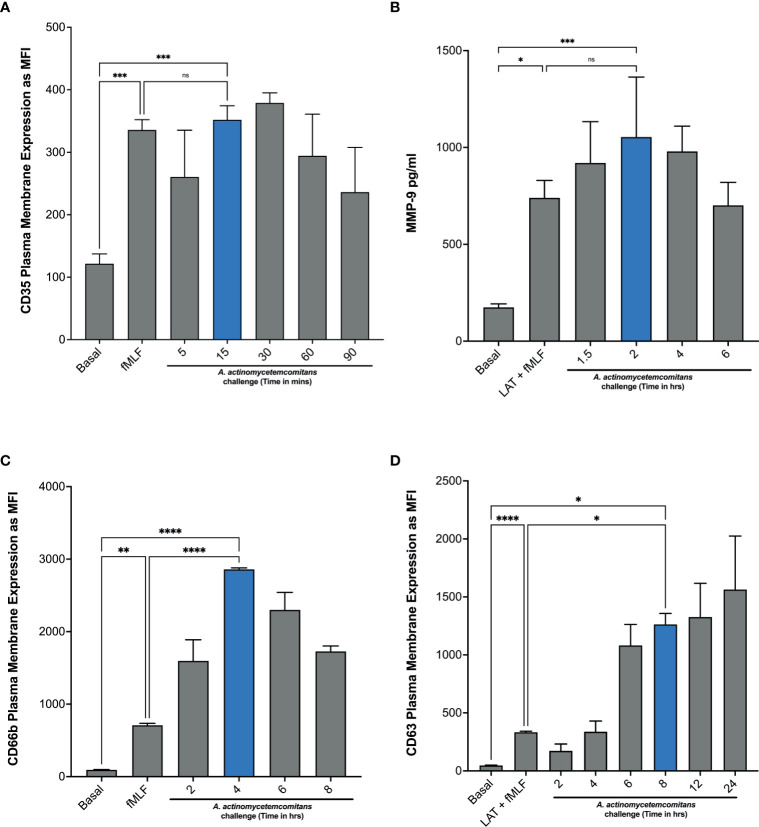
*A*. *actinomycetemcomitans* induces exocytosis of all four neutrophil granule subtypes. Induction of neutrophil granule exocytosis was determined by challenging human neutrophils with *A*. *actinomycetemcomitans* (MOI 50) for 1.5 h, 6 h, 8 h, and 24 h Increased plasma membrane expression of CD35 **(A)**, CD66b **(C)**, and CD63 **(D)** was measured to define secretory vesicles, specific granules, and azurophil granule exocytosis, respectively, by flow cytometry. Basal levels and latrunculin (Lat) + fMLF were used as negative and positive control. Granule markers are plotted as the mean channel florescence intensity (MFI) ± SEM of three independent experiments. For gelatinase granule exocytosis, cell supernatants were collected at basal and following each stimulation, and levels of matrix metallopeptidase 9 (MMP-9) were measured by ELISA **(B).** MMP-9 concentration is plotted as the mean ± SD of three independent experiments. Statistical differences among experimental conditions and time points were analyzed by repeated measures one-way ANOVA, followed by Tukey’s post-test. ns, not significant, *p < 0.05, **p < 0.01, ***p < 0.001, ****p < 0.0001.

In **Figure 1**, we showed that treatment with latrunculin A followed by stimulation with fMLF, which is known to induce the exocytosis of gelatinase and azurophilic granules, induced epinephrine release by human neutrophils, suggesting that epinephrine may be stored in one or more of the neutrophil granules. To demonstrate this, we first determined the ability of *Aa* to induce granule exocytosis. Neutrophils were challenged with *Aa* and granule exocytosis was determined by measuring increase of plasma membrane expression of secretory vesicles, specific granules, and azurophilic granule markers by flow cytometry (see gating strategy and histograms; **Supplementary Figure 4**) and the extracellular release of MMP-9 by ELISA. Stimulation with *Aa* induced significant secretory vesicles mobilization by 15 min, as indicated by increased CD35 expression (**Figure 3A**), which was similar to the exocytosis induced by the positive control fMLF. In addition, gelatinase granule exocytosis, measured by ELISA, was significantly induced after 2 h of *Aa* challenge (**Figure 3B**). Specific granule exocytosis, measured by increased CD66b expression, was significantly increased after 4 h of *Aa* challenge (**Figure 3C**). Azurophilic granule exocytosis, measured by increased CD63 expression, was significantly increased starting at 8 h post bacterial challenge (**Figure 3D**). Mobilization of azurophilic granules requires stronger stimulation compared to the other granule subtypes; our results showed that these granules continued to be mobilized by *Aa* increasingly up to 24 h (**Figure 3D**). These results demonstrate that *Aa* challenge induced exocytosis of all four neutrophil granule subtypes.

**Figure 4:**
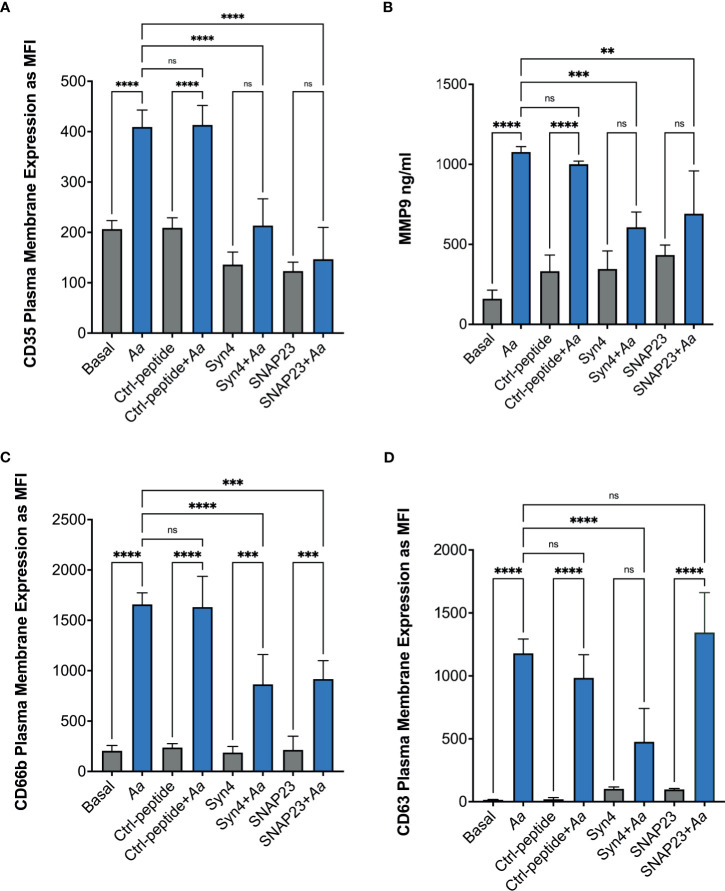
Pre-treatment with TAT fusion proteins prevented *A. actinomycetemcomitans-*induced neutrophil granule exocytosis. Human neutrophils were unstimulated, challenged with *A*. *actinomycetemcomitans (Aa)* at MOI 50, or pre-treated with TAT-Syntaxin 4 (Syn4), TAT-SNAP23 (SNAP23), and TAT-Control peptide (Ctrl-peptide) for 15 min followed by *A*. *actinomycetemcomitans* challenge. The peak time point of *Aa*-induced granule exocytosis was different for each granule subtype. Secretory vesicle peak was at 15 min post-bacterial challenge **(A)**, 2 h for gelatinase granules **(B)**, 4 h for specific granules **(C)**, and 8 h for azurophilic granules **(D)**. Granule markers were measured by flow cytometry and plotted as the mean channel florescence intensity (MFI) ± SEM of four independent experiments **(A, C, D)**. For gelatinase granules, matrix metallopeptidase 9 (MMP-9) was measured from human neutrophil supernatant by ELISA and MMP-9 concentration is plotted as the mean ± SD of four independent experiments **(B)**. Statistical differences among experimental conditions were analyzed by ordinary one-way ANOVA, followed by Tukey’s post-test. ns, not significant, **p < 0.01, ***p < 0.001, ****p < 0.0001.

To further characterize if epinephrine release by neutrophils was caused by *Aa* inducing granule exocytosis, we took advantage of two degranulation inhibitors, TAT-SNAP23, which selectively inhibits secretory vesicles, gelatinase granules, and specific granules (31), and TAT-Syntaxin 4, which blocks exocytosis of all four granule subtypes (40). Human neutrophils were pre-treated with TAT-Syntaxin 4, TAT-SNAP23, or TAT-control peptide fusion proteins, followed by *Aa* challenge at 15 min, 2 h, 4 h, and 8 h. Granule exocytosis was measured by increased plasma membrane expression of CD35 (secretory vesicles), CD66b (specific granules), and CD63 (azurophilic granules) markers by flow cytometry and the extracellular release of MMP-9 by ELISA. Treatment with TAT-Syntaxin 4 reduced *Aa*-induced mobilization of secretory vesicles (**Figure 4A**), gelatinase granules (**Figure 4B**), specific granules (**Figure 4C**), and azurophilic granules (**Figure 4D**). The *Aa*-induced mobilization of secretory vesicles, gelatinase, and specific granules (**Figures 4A–C**) was significantly reduced by TAT-SNAP23 pre-treatment. As expected, TAT-SNAP23 failed to inhibit *Aa*-induced azurophilic granule exocytosis (**Figure 4D**). Furthermore, cell supernatants were collected at each time point, and granule content release of albumin, lactoferrin, and myeloperoxidase were measured by ELISA. Similar to the results obtained by flow cytometry, challenge of neutrophils with *Aa* for 15 min induced significant release of albumin, which was significantly reduced by pre-treatment with TAT-SNAP23 and TAT-Syntaxin 4, but not TAT-control (**Supplementary Figure 5A**). Similarly, *Aa* challenge for 4 h induced release of lactoferrin, and its release was successfully reduced by TAT-SNAP23 and TAT-Syntaxin 4, but not TAT-control (**Supplementary Figure 5B**). As expected, neutrophils challenged with *Aa* for 8 h resulted in significant release of myeloperoxidase, which was inhibited by TAT-Syntaxin 4 but not TAT-SNAP23 or TAT-control (**Supplementary Figure 5C**).

**Figure 5:**
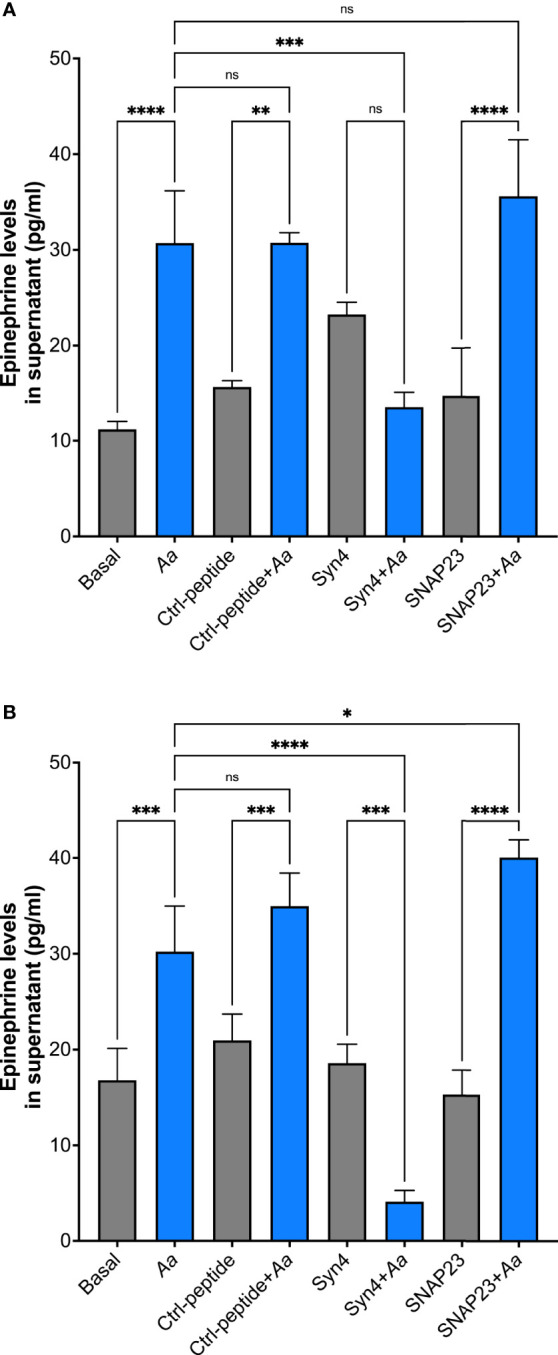
Human neutrophils store epinephrine in azurophilic granules. Epinephrine storage location was determined by pre-treating human neutrophils with TAT-Syntaxin 4 (Syn4), TAT-SNAP23 (SNAP23), or TAT-Ctrl (Ctrl) for 15 min, followed by a 4-h challenge with *A*. *actinomycetemcomitans* at MOI 50 **(A)** or 8 h **(B)**. Epinephrine concentrations are plotted as the mean ± SD of three independent experiments. Statistical differences among experimental conditions were analyzed by ordinary one-way ANOVA, followed by Tukey’s post-test. ns, not significant, *p < 0.05, **p < 0.01, ***p < 0.001, ****p < 0.0001.

To further characterize which granule subtype might store epinephrine, supernatants were collected to measure epinephrine levels by ELISA. *A. actinomycetemcomitans* challenge of human neutrophils at 15 min showed no significant release of epinephrine similar to basal and TAT-control (**Supplementary Figure 6A**). Moreover, *Aa* challenge at 2 h induced significant release of epinephrine in human neutrophils; pre-treatment of TAT-Syntaxin 4 was able to inhibit epinephrine release but TAT-SNAP23 failed to inhibit release (**Supplementary Figure 6B**). On the other hand, only pre-treatment with TAT-Syntaxin 4, but not TAT-SNAP23, blocked *Aa*-induced epinephrine released after 4 h (**Figure 5A**) and 8 h (**Figure 5B**) of bacterial challenge. Since TAT-SNAP23 does not inhibit azurophilic granule exocytosis, these results demonstrate that epinephrine is stored in azurophilic granules. Furthermore, this shows that epinephrine release by neutrophils is linked to granule exocytosis.

### *A. actinomycetemcomitans* Interacts With Epinephrine in a QseC-Dependent Manner

**Figure 6:**
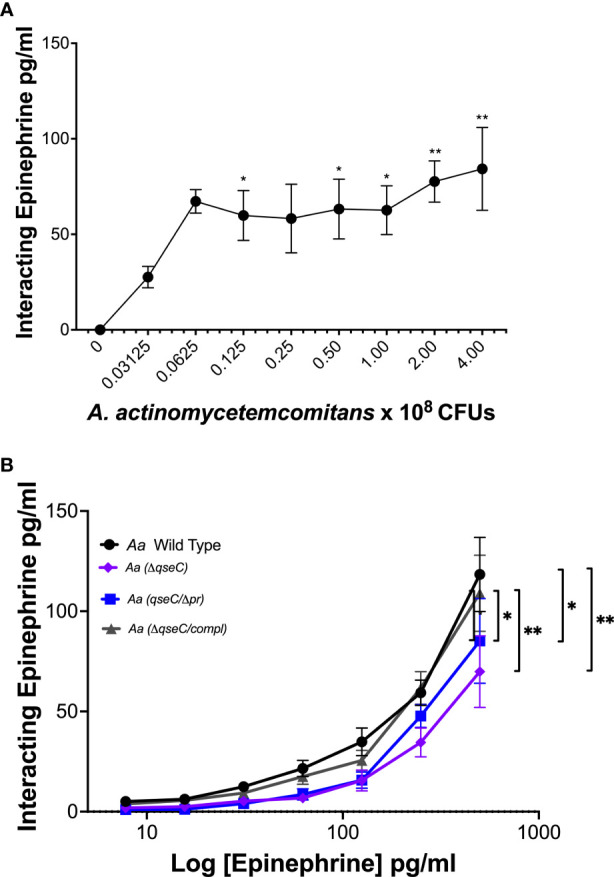
*A*. *actinomycetemcomitans* interacts with epinephrine in a QseC-dependent manner. The interaction of epinephrine and *A*. *actinomycetemcomitans* was determined by incubation of increasing colony-forming units (CFUs) of *A*. *actinomycetemcomitans* with 250 pg/ml of recombinant epinephrine. Associated epinephrine was plotted against increasing CFUs of *Aa* **(A)**. Requirement of QseC for epinephrine interaction with *A. actinomycetemcomitans* was determined by incubation of the bacterium with increasing concentrations of recombinant epinephrine (0–500 pg/ml) **(B)**. Epinephrine levels were measured by ELISA and plotted as the mean ± SD of five independent experiments. Statistical differences among experimental conditions were analyzed by ordinary one-way ANOVA **(A)** or two-way ANOVA **(B)**, followed by Tukey’s post-test. *p < 0.05. **p < 0.01.

To determine if *Aa* interacts with epinephrine, increasing CFUs of *Aa* were incubated with a fixed concentration of recombinant epinephrine. Epinephrine interaction with *Aa* increased with increasing *Aa* CFUs (**Figure 6A**). Previous work in our lab demonstrated that QseBC was required for *Aa* biofilm growth and virulence (25) and that the periplasmic domain of QseC was required for activation of the TCS by catecholamines and iron (6). Therefore, we performed epinephrine dose interaction assays using various *Aa* strains deficient for QseC. Interaction of epinephrine was diminished by deletion of the QseC sensor (*ΔqseC)* and in the strain expressing QseC with an in-frame deletion of the periplasmic domain (*qseCΔp)* (**Figure 6B**). The ability of epinephrine to interact with *Aa* was restored when the *qseC* gene was complemented into the QseC deletion mutant (*ΔqseC-comp* **)** (**Figure 6B**). These results show that *Aa* interaction with epinephrine requires QseC and is dependent on the QseC periplasmic domain.

### Epinephrine From Human Neutrophils Promotes *A. actinomycetemcomitans* Growth and *qseBC* Expression

**Figure 7:**
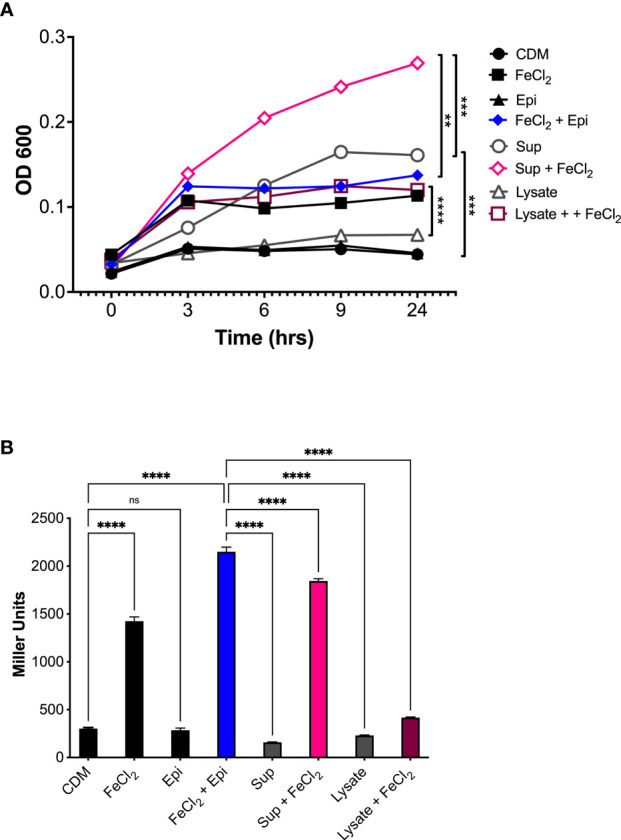
Host-derived epinephrine promotes *A*. *actinomycetemcomitans* growth and *qseBC* expression. The supernatants and lysates from human neutrophils stimulated with latrunculin A and fMLF were collected and used as supplement for chemically defined media (CDM). *A*. *actinomycetemcomitans* was grown under anaerobic conditions in CDM alone or supplemented with human neutrophil supernatant (Sup), cell lysate (Lysate), iron (FeCl_2_, 100 μM), or Epinephrine (Epi, 50 μM). At designated times, growth was measured by determining the optical density at 600 nm (OD_600_) **(A)**. *A*. *actinomycetemcomitans* strain (that harbors the *qseBC* promoter-*lacZ* reporter plasmid pDJR29) was grown under anaerobic conditions in CDM alone or supplemented with human neutrophil supernatant (Sup), cell lysate (Lysate), iron (FeCl_2_, 100 μM), or Epinephrine (Epi, 50 μM). Expression of the *qseBC* operon was determined by measuring β-galactosidase activity after 24 h **(B)**. The OD_600_ measurements for growth were plotted as the mean ± SEM of three independent experiments **(A)**. The expression of the *qseBC* operon is presented in miller units as the mean ± SD of three independent experiments **(B)**. Statistical differences among experimental conditions were analyzed by two-way ANOVA **(A)** or ordinary one-way ANOVA **(B)**, followed by Tukey’s post-test. ns, not significant, **p < 0.01, ***p < 0.001, ****p < 0.0001.

To determine if the epinephrine levels released by human neutrophils could promote bacterial growth, the supernatants and cell lysates were collected from cells stimulated with latrunculin A and fMLF and used to assess *Aa* growth and expression of the *qseBC* operon. *A. actinomycetemcomitans* was grown anaerobically in CDM alone or supplemented individually or in combination with neutrophil supernatant, neutrophil cell lysate, iron (100 μM), or epinephrine (50 μM). Growth in CDM supplemented with neutrophil lysate or epinephrine alone was comparable to the unsupplemented CDM control (**Figure 7A**). Supplementation with iron, either alone or in combination with epinephrine, resulted in a significant induction of *Aa* growth (**Figure 7A**). Furthermore, medium supplemented with neutrophil supernatant alone promoted growth, which was increased when supplemented with iron (**Figure 7A**). On the other hand, neutrophil cell lysate supplemented with iron promoted growth similar to epinephrine + iron or neutrophil supernatant alone (**Figure 7A**). Supplementation with epinephrine, neutrophil supernatant, or lysate alone failed to induce expression of QseBC (**Figure 7B**). QseBC expression was significantly induced by iron and a further significant increase in expression occurred by supplementation with a combination of iron with epinephrine or neutrophil supernatant (**Figure 7B**). Unexpectedly, supplementation with iron and neutrophil lysate suppressed QseBC expression (**Figure 7B**). This experiment was performed under aerobic conditions with similar results (data not shown). Here, we show that *Aa* makes use of neutrophil-derived epinephrine to promote bacterial growth and expression of the QseBC in the anaerobic environment.

## Discussion

The newly emerging field of microbial endocrinology (16, 44) studies the communication or inter-kingdom signaling (45) that has evolved between microorganisms and their hosts. The study of inter-kingdom signaling includes the study of hormonal communication, where microorganisms respond to the host neurohormones as environmental cues to regulate the expression of genes necessary for virulence and survival (44, 46–49). There are reports of host–pathogen crosstalk involving *Aa* biofilm sequestering and taking up IL-1β (50). Additionally, it has been shown previously that *Aa* expresses a cytokine binding receptor, BilRI, that allows it to benefit from cytokine release to promote biofilm formation and bacterial growth (51, 52). Previous work by our group demonstrated that the presence of catecholamines and iron *in vitro* promoted *Aa* growth and regulated the expression of genes necessary for virulence and survival in the anaerobic environment (6). However, those studies did not determine if similar effects occurred *in vivo*. Based on work by others, it is known that there is a large infiltration of neutrophils at the subgingival pocket during periodontitis (26, 53, 54) and murine neutrophils have been shown to release and synthesize catecholamines when stimulated with LPS (27). Therefore, we considered human neutrophils as a potential source of catecholamines for *Aa*. In our *ex vivo* studies, we showed that *Aa* induces epinephrine release in human neutrophils depleting stored catecholamine levels and indirectly inducing catecholamine synthesis. As a novel finding, we identified azurophilic granules as the storage location of epinephrine in human neutrophils and that *Aa* gains access to epinephrine by inducing granule exocytosis. Finally, we proved that host-derived epinephrine promoted *Aa* growth and QseBC expression under anaerobic conditions.

Marino et al. (28) detected catecholamines in human peripheral blood mononuclear cell medium, but the report failed to determine if these catecholamines were released and, if so, what stimuli induced release. Likewise, catecholamine release has been shown in other immune cells such as rat and human lymphocytes (55, 56). Although there is extensive work done in murine neutrophils, there was no report of human neutrophils releasing catecholamines and identified stimuli. Here, we present for the first time that human neutrophils release epinephrine when stimulated with latrunculin A + fMLF and when challenged with *Aa*, but not by *F. alocis*, which occupies the same oral niche as *Aa* (see below). These observations support previous findings by Parantainen et al. (39), the first to identify endogenous catecholamines in human neutrophils. However, at the time, the source of these catecholamines was not determined or if neutrophils participated in catecholamine synthesis. Later, further evidence of catecholamines present intracellularly in human neutrophils was found and enzymes involved in catecholamine metabolism were detected by high-performance liquid chromatography (HPLC) (57). In our experiments, we observed that the levels of epinephrine in cell lysates increased after 8 h and 24 h of bacterial challenge, suggesting *de novo* catecholamine synthesis by human neutrophils. This was confirmed by the presence of significant levels of tyrosine hydroxylase levels in the presence of *Aa*. In contrast, dopamine β-hydroxylase levels decreased when *Aa* was present. Tyrosine hydroxylase catalyzes the first and rate-limiting step of the catecholamine synthesis pathway, explaining larger amounts of this enzyme. A potential reason for this is that dopamine β-hydroxylase catalyzes dopamine into norepinephrine, and not all dopamine will be catalyzed, explaining the decrease in enzyme levels. We also found significant levels at 4 h of the catecholamine inactivating enzymes catechol-o-methyltransferase and monoamine oxidase-A. This correlates with the increased epinephrine release induced by *Aa* at 4 h; as epinephrine is released, demand for degradation increases. Similarly, as stored epinephrine is depleted, there is demand for catecholamine synthesis at later time points, and inactivation enzymes decrease at this time. A potential limitation of our study is that granule exocytosis experiments were done under aerobic conditions and not under low oxygen or anaerobic conditions. The periodontal pocket is considered a hypoxic environment, with decreasing oxygen levels as the depth of the periodontal pocket increases, becoming increasingly hypoxic during infection due to activation of the NADPH oxidase complex (58, 59). Therefore, it is likely that neutrophils are exposed to hypoxic conditions *in vivo* when recruited to the gingival pocket. Although we did not perform experiments with both *Aa* and neutrophils under anaerobic conditions, preliminary work in our lab with neutrophils challenged with *F. alocis* showed no difference in the lifespan of neutrophils grown aerobically or anaerobically. Additionally, untreated neutrophils under anaerobic conditions resulted in enhanced viability compared to cells grown aerobically. Though we infer that human neutrophils will undergo granule exocytosis under hypoxic conditions, this remains to be tested in the future.

We demonstrated that *Aa* induces the mobilization of secretory vesicles, gelatinase granules, specific granules, and azurophilic granules in human neutrophils. These findings align with previous observations that identified enhanced surface expression of CD63 and CD66b in oral neutrophils, characteristic of neutrophils that are undergoing active granule exocytosis (60, 61). Johansson et al. (62) showed that purified leukotoxin A induces release of granule contents from human neutrophils, and the authors observed increased expression of CD63 and CD66b along with the release of elastase and lactoferrin. In our *ex vivo* model, we obtained similar observations by challenging human neutrophils with a low leukotoxic strain of *Aa* compared to high concentrations of purified leukotoxin. Furthermore, through selective inhibition of granule exocytosis, we identified azurophilic granules as the storage location for epinephrine in human neutrophils. To the best of our knowledge, this observation serves as the first evidence of the storage location of epinephrine in human neutrophils. Human neutrophils are recruited out of circulation by interaction with neutrophil-specific adhesion molecules on the blood vessel wall. This interaction in addition to chemoattractant gradient such as IL-8 promotes the mobilization of secretory vesicles. Once neutrophils cross through the epithelium, they continue to receive signals such as increasing IL-8 gradient potentially released by gingiva epithelial cells and formyl peptides from invading bacteria, resulting in mobilization of gelatinase granules. The primed neutrophil arrives at the infected subgingival pocket where there are overwhelming inflammatory signals that may induce the release of specific and azurophilic granules. Alternatively, these granules can fuse with vacuoles containing phagocytized bacteria, forming a phagolysosome (63–65). Thus, *Aa* may induce the release of azurophilic granules at the subgingival pocket to gain access to epinephrine and in turn the release of potent antimicrobial protein contents of these granules may contribute to tissue damage and disease progression.

When human neutrophils were challenged with *Aa* for 2 h, we expected to see a high release of epinephrine, due to depleted epinephrine levels in cell lysate. A plausible explanation to the difference in levels of epinephrine released versus stored at 2 h is that some of the epinephrine released could be interacting with *Aa* and therefore no longer in the supernatant. Epinephrine interaction with *Aa* increased with increasing *Aa* CFUs. Furthermore, *Aa* interaction with epinephrine was found to require QseC, in a QseC periplasmic domain-dependent manner. This supports previous findings made by our group that identified catecholamines (norepinephrine and epinephrine) as the signal that is sensed by QseC and that the periplasmic domain of QseC is required for *qseBC* operon expression in the presence of catecholamines and iron (6). It also strengthens the importance of QseC in recognizing catecholamines for growth and virulence of *Aa* (25). The exact details of the interaction of *Aa* with epinephrine remains unclear, but the discussed results make us conclude that the QseC periplasmic domain is required. As for a receptor for epinephrine, when *Aa* is grown in the presence of catecholamines and iron, the expression of the enterobactin operon, which contains a gene for FepA, remains unchanged (6). In *E. coli*, FepA is an outer membrane protein that serves as a receptor for the ferric enterobactin siderophore (66, 67). The uptake of this siderophore into the bacterial cells is TonB-dependent (67, 68). We performed different experiments to determine if FepA was required for *Aa* interaction with epinephrine using a *ΔfepA* mutant *Aa* strain and we found no difference in interaction compared with the wild-type strain (data not shown). Moreover, as an alternative for iron entry into the bacterial cells, there are the ferrous iron transport system (Feo) and periplasmic-binding protein-dependent transport (PBT) (69–72). These are predominant iron transport systems at low oxygen conditions when ferrous iron is stable and is more prevalent than ferric iron (17). In the case the ferric iron is predominant, it will be chelated by ferric iron reductases and then uptake is mediated by ferrous permeases (73, 74). Rhodes et al. (19) demonstrated that *Aa* makes use of inorganic iron to grow under chelated conditions and proposed that *Aa* may acquire iron through the expression of systems which function independently of an outer membrane receptor or TonB-dependent transport. The elucidation of which TonB and receptor independent iron transport system is used by *Aa* will require further investigation.

Under anaerobic conditions, we showed that *Aa* growth in limited media is promoted in the presence of neutrophil supernatant or cell lysate supplemented with iron. Likewise, supplementation with neutrophil supernatant and iron induced expression of QseBC. Interestingly, neutrophil cell lysate alone or supplemented with iron QseBC expression was suppressed. Human neutrophil lysates contain all internal contents of neutrophils, including components such as calprotectin. Calprotectin is a metal binding protein, abundant in neutrophil cytoplasm (75, 76). Another protein that is found in specific granules is the neutrophil gelatinase-associated lipocalin. This protein scavenges iron from siderophores, and it has been shown to remove iron from catecholamines as well (77). These proteins can be sequestering the supplemented iron competing with epinephrine present in lysate, making it unavailable to *Aa* and consequentially suppressing the induction of the QseBC.

In *Aa*-positive subjects, *F. alocis* was detected at high levels (42) and *F. alocis* accumulation in the oral biofilm has been shown to be stimulated by the presence of specific strains of *Aa* (43). Based on our experiments, we determined that *F. alocis* does not induce release of epinephrine in human neutrophils. This result goes in hand with previous findings that *F. alocis* does not induce the exocytosis of azurophilic granules (38, 78). In the case that *F. alocis* is found to be catecholamine-responsive, it will suggest that it may be benefiting from the ability of *Aa* to induce epinephrine release. However, at present, there is no report of *F. alocis* being influenced in some way by catecholamines. *A. actinomycetemcomitans* has also been found to be associated to *Fusobacterium nucleatum*, whose growth is increased by hormones like epinephrine and norepinephrine (21); *Lactobacillus* spp. expresses transporter systems for uptake of catecholamines (79) and other catecholamine-responsive species such as *Prevotella* spp. and *Leptotrichia* (16, 80, 81). Some of these bacteria have been shown to lack the ability to produce siderophores as is the case of *Aa* (82, 83). Therefore, it is likely that these bacteria work as a team to thrive in the subgingival pocket, where the role of *Aa* may be to induce catecholamine (i.e., epinephrine) release by the infiltrating human neutrophils.

In conclusion, our *ex vivo* experiments demonstrated that human neutrophils store and release epinephrine upon stimulation with latrunctin A + fMLF. We show that *Aa* can induce epinephrine release in human neutrophils, consequently depleting epinephrine storage and promoting catecholamine metabolism. Furthermore, one of the most important contributions of this study was the finding of azurophilic granules as the storage location of epinephrine in human neutrophils and that *Aa* gains access to it by inducing granule exocytosis. Additionally, we found that epinephrine interaction with *Aa* depends on QseC expression and requires the QseC periplasmic domain. Finally, human-neutrophil-derived epinephrine supplemented with iron promoted *Aa* growth and QseBC expression under anaerobic conditions. The presented findings supply evidence towards the growing field of microbial endocrinology by contributing to the understanding of the crosstalk between bacteria and the host endocrine system. Furthermore, it expands the knowledge of the role of stress hormones in periodontal disease and potentially other chronic inflammatory diseases.

## Data Availability Statement

The raw data supporting the conclusions of this article will be made available by the authors, without undue reservation.

## Ethics Statement

The studies involving human participants were reviewed and approved by Institutional Review Board of the University of Louisville. The patients/participants provided their written informed consent to participate in this study.

## Author Contributions

HO performed the experiments and participated in the study design, data analysis and interpretation, and drafting of the manuscript. SU and DD equally participated in the study design, data interpretation, critical revision of the manuscript, and study supervision. DD obtained funding for the study. All authors contributed to the article and approved the submitted version.

## Funding

This work was supported by the Integrated Programs in Biomedical Sciences Graduate Fellowship at the University of Louisville School of Medicine, by the National Institute of Dental and Craniofacial Research (NIDCR) grants RO1DE014605 (DD/SU) and R01DE024509 (SU), and by the Ruth L. Kirschstein National Research Service Award by the NIDCR F31DE027585 (HO).

## Conflict of Interest

The authors declare that the research was conducted in the absence of any commercial or financial relationships that could be construed as a potential conflict of interest.

## Publisher’s Note

All claims expressed in this article are solely those of the authors and do not necessarily represent those of their affiliated organizations, or those of the publisher, the editors and the reviewers. Any product that may be evaluated in this article, or claim that may be made by its manufacturer, is not guaranteed or endorsed by the publisher.
