# Supplementary material for: The Hunger Games: Aggregatibacter actinomycetemcomitans Exploits Human Neutrophils As an Epinephrine Source for Survival
Source: Front Immunol. 2021 Aug 12;12:707096. doi: 10.3389/fimmu.2021.707096 (PMC8387626; doi:10.3389/fimmu.2021.707096)
Supplement: Supplementary file 1 [file DataSheet_1.docx]

Supplementary Material

# Supplementary Tables

**Table S1. Strains used in this work**

| **Strain** | **Derived/characteristics*** | **Source/Reference** |  |
| --- | --- | --- | --- |
|  |  |  |  |
| ***Aggregatibacter actinomycetemcomitans*** | | |  |
| 652 | Wild type, serotype c (Vanco^r^, Baci^r^) | Laboratory stock |  |
| 652-JR23 | *qseC*Δp (Sp^r^) | Juárez-Rodríguez *et al*. (2013) |  |
| 652-JR38 | Δ*qseC* (Sp^r^) | Juárez-Rodríguez *et al*. (2013) |  |
| 652-JR38 | Δ*qseC-comp* (Sp^r^) | Juárez-Rodríguez *et al*. (2013) |  |
| 652(pDJR29) | Km^r^, *ygiW-qseBC* promoter _-1 to -372_-*lacZ* | Juárez-Rodríguez *et al*. (2013) |  |
| ^*^ Vanco^r^ – vancomycin resistance, Baci^r^ – bacitracin resistance, Km^r^ - kanamycin resistance, Sp^r^ - spectinomycin resistance | | |  |

**Table S2: Composition of chemically defined media (CDM)**

| **Amino Acid Stock** | | | | | | | |
| --- | --- | --- | --- | --- | --- | --- | --- |
| **Hydrophilic Amino Acids** | | **Hydrophobic Amino Acids** | | **Purine/Pyrimidine** | | **L-cystine** | |
| 10mL | H_2_O | 15mL | H_2_O | 10mL | H_2_O | 5mL | H_2_O |
| 2.5mL | 2M NaOH | 3.125mL | 2M NaOH | 1.25mL | 2M NaOH | 0.625mL | 2M NaOH |
| 499.2mg | L-glutamic Acid | 200mg | L-alanine | 25mg | Adenine | 10mg | L-cystine |
| 200mg | Glycine | 200mg | D-alanine | 20mg | Guanine |  |  |
| 200mg | L-Threonine | 200mg | L-leucine | 27mg | Cytosine hydrochloride |  |  |
| 200mg | L-Serine | 200mg | L-valine | 20mg | Thymine |  |  |
| 200mg | L-lysine hydrochloride | 200mg | L-Tryptophan | 20mg | Xanthine |  |  |
| 242mg | L-arginine hydrochloride | 200mg | L-methionine | 20mg | Hypoxanthine |  |  |
| 270.2mg | L-histidine hydrochloride | 200mg | L-isoleucine | 20mg | Uracil |  |  |
| 200mg | L-glutamine | 200mg | L-phenylalanine |  |  |  |  |
| 227.2mg | L-asparagine | 40mg | L-tyrosine |  |  |  |  |
| 200mg | L-proline |  |  |  |  |  |  |
| 200mg | L-aspartic acid |  |  |  |  |  |  |
| 40mg | L-ornithine hydrochloride |  |  |  | |  |  |
| 40mg | L-hydroxyproline |  |  |  |  |  |  |
| Combine hydrophobic, hydrophilic, purine & pyrimidine and L-cystine to make amino acid stock. | | | | | | | |
|  |  |  |  |  |  |  |  |
| **Inorganic Salt Stock** | | **Vitamin Stock** | | **Pimelic Acid/Biotin** | | **1L CDM** | |
| 99.5mL | H_2_O | 20mL | H_2_O | 5mL | H_2_O | 10mL | CaCl_2_ (10mg/mL) |
| 10mg | MnSO_4_ | 1000mg | Choline chloride | 5mL | Ethanol | 50mL | Amino Acid Stock |
| 200mg | NaCl | 200mg | β-alanine | 1mg | Pimelic Acid | 50mL | Inorganic Salt Stock |
| 400mg | K_2_HPO_4_ | 20mg | Pyridoxal | 1mg | D-Biotin | 1mL | Vitamin Stock |
| 2000mg | KH_2_PO_4_ | 20mg | Pyridoxine-HCl | **Lipoamide** | | 50mL | NaHCO_3_ (20mg/mL) |
| 200mg | KNO_3_ | 20mg | Pyridoxamine-di-HCl | 1mL | β-mercaptoethanol | 10mL | Cysteine (65mg/mL) |
| 0.1mg | KI | 20mg | Spermidine-tri-HCl | 9mL | Ethanol | 10mL | MgSO_4_ (70mg/mL) |
| 0.065mg | CuSO_4_:5H_2_O | 20mg | Nicotinic Acid | 1mg | Lipoamide | 1mL | Pimelic acid/Biotin |
| 0.5mg | Boric Acid | 20mg | Calcium pantothenate |  |  | 10mL | Riboflavin (0.1mg/mL) |
| 0.7mg | ZnSO_4_:7H_2_O | 20mg | Spermine-tetra-HCl | **Folic Acid** | | 1mL | Lipoamide |
| 0.5mg | Sodium molybdate | 20mg | Thiamine-HCl | 9.975mL | H_2_O | 1mL | Folic Acid |
|  |  | 200mg | Myo-Inositol | 25uL | NH_4_OH |  |  |
|  |  | 20mg | NAD | 10mg | Folic Acid |  |  |
|  |  | 2mg | *p*-aminobenzoic acid |  |  |  |  |
|  |  | .00005mg | Vitamin B12 |  |  |  |  |

# Supplementary Figures


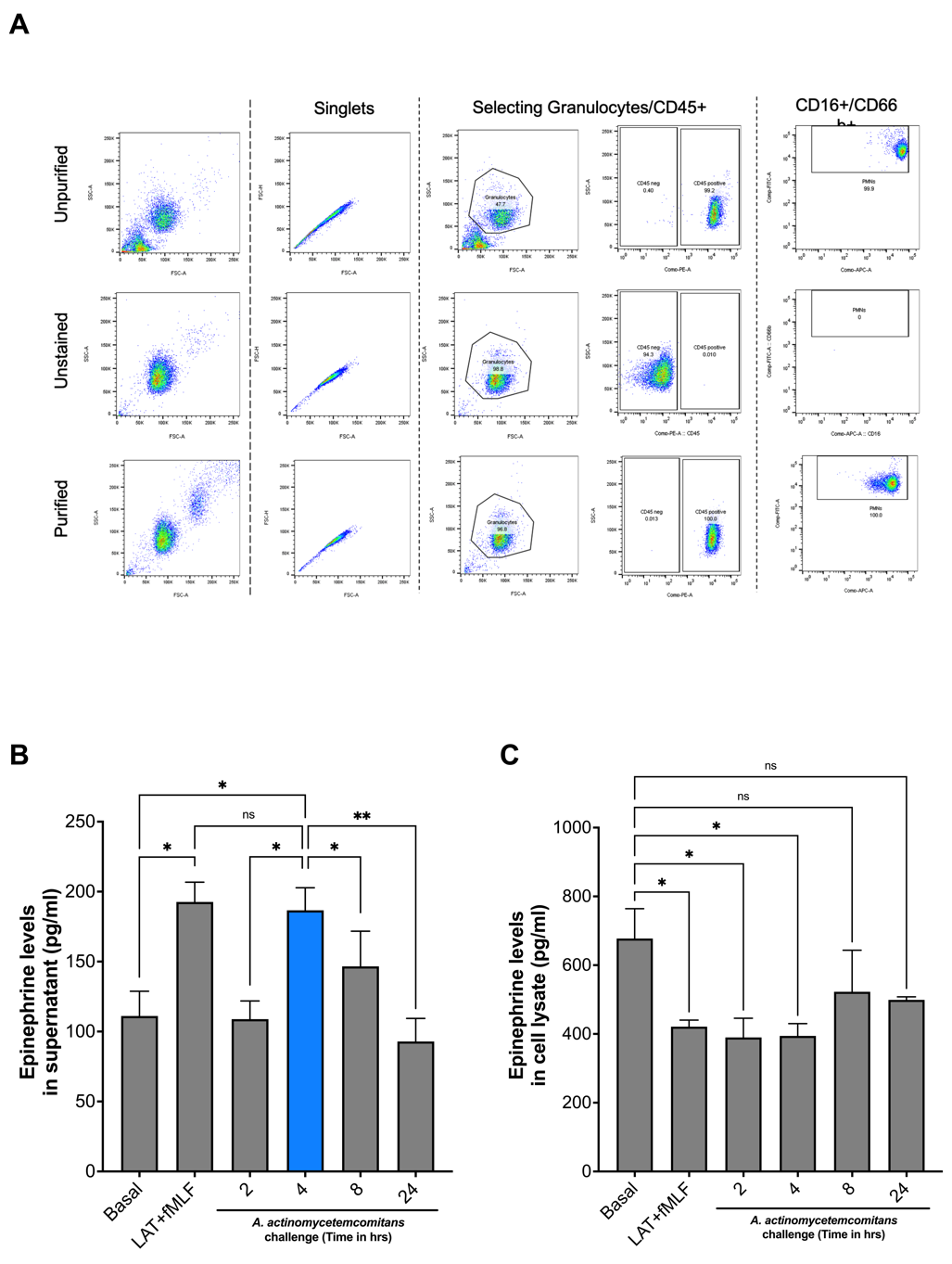


**Figure S1. *A. actinomycetemcomitans* induces epinephrine release in highly purified human neutrophils**. Isolated human neutrophils were highly purified (see Methods) starting from a >90-95% pure neutrophil isolation, confirmed by flow cytometry staining using the gating strategy shown in panel A (A). and then challenged with *A. actinomycetemcomitans* (MOI 50) for 2h, 4h, 8h and 24h and epinephrine content was measured by ELISA from collected supernatant **(**B**)** and cell lysate **(**C**)**. Epinephrine concentration is plotted as the mean ± SD of 3 independent experiments. Statistical differences among experimental conditions and time points were analyzed by repeated measures one-way ANOVA, followed by Tukey’s post-test. ns: not significant, **p* < 0.05, ***p* < 0.01.

**
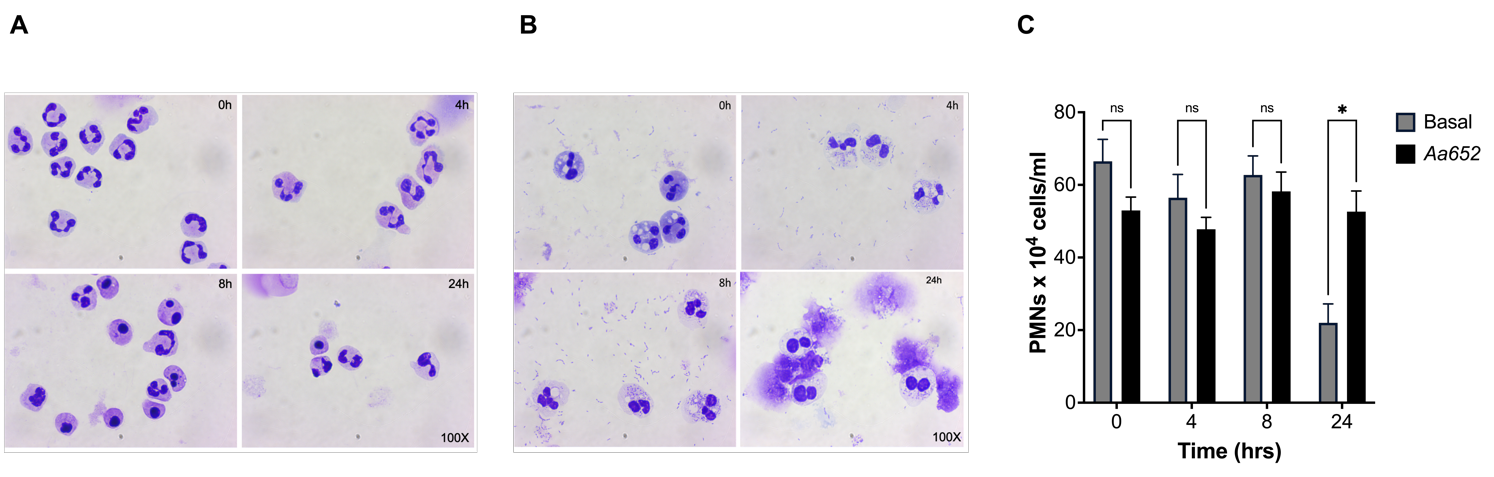
**

**Figure S2. Human neutrophils are viable after 24h exposure to *A. actinomycetemcomitans.*** Neutrophil viability when challenged with *A. actinomycetemcomitans* was determined by cytospin imaging **(A-B)** and Trypan Blue exclusion **(C)**. Human neutrophils were challenged with *A. actinomycetemcomitans* (MOI 50) for 0h, 4h, 8h, 24h, centrifuged and washed with RPMI-1640. Neutrophils were suspended at 1 x 10^5^ cells in RPMI-1640 and human serum. The cell suspension was loaded into the funnel chamber and assembled on the cytocentrifuge clip, with slide and filter. Cytocentrifuge clip was centrifuged, and the microscope slide was removed from cytocentrifuge clip and fixed and stained. Images of unchallenged neutrophils **(A)** and *A. actinomycetemcomitans* challenged neutrophils **(B)** are shown. Neutrophils were diluted 1:20 in Trypan blue at 0h, 4h, 8h, 24h and live cells were counted using a hemacytometer **(C)**. Viability counts are plotted as the mean ± SD of 3 independent experiments. Statistical differences among time points were analyzed by two-way ANOVA, followed by Bonferroni’s post-test. ns: not significant, **p* < 0.05.

**
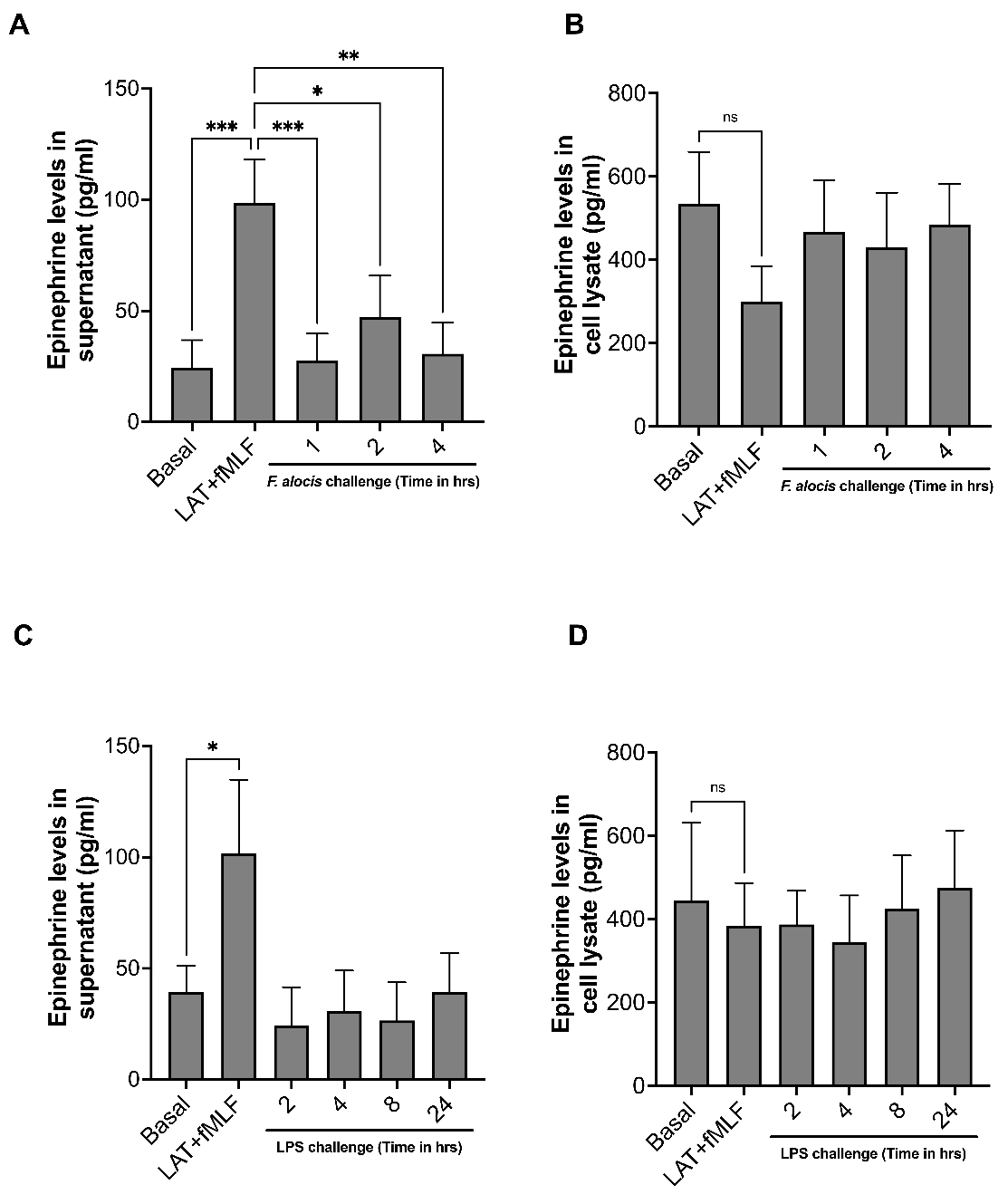
**

**Figure S3. *F. alocis* is unable to induce epinephrine release in human neutrophils.** Human neutrophils were challenged with *F. alocis* (MOI 10) for 1h, 2h and 4h or with LPS for 1h, 2h, 4h, 8h and 24h. Epinephrine content was measured by ELISA from collected supernatant and cell lysate of *F. alocis* (A-B) challenged neutrophils and supernatant and cell lysate of neutrophils treated with LPS (C-D). Epinephrine concentration is plotted as the mean ± SD of 5 independent experiments. Statistical differences among experimental conditions and time points were analyzed by repeated measures one-way ANOVA, followed by Tukey’s post-test. ns: not significant, **p* < 0.05, ***p* < 0.01, ****p* < 0.001.

**
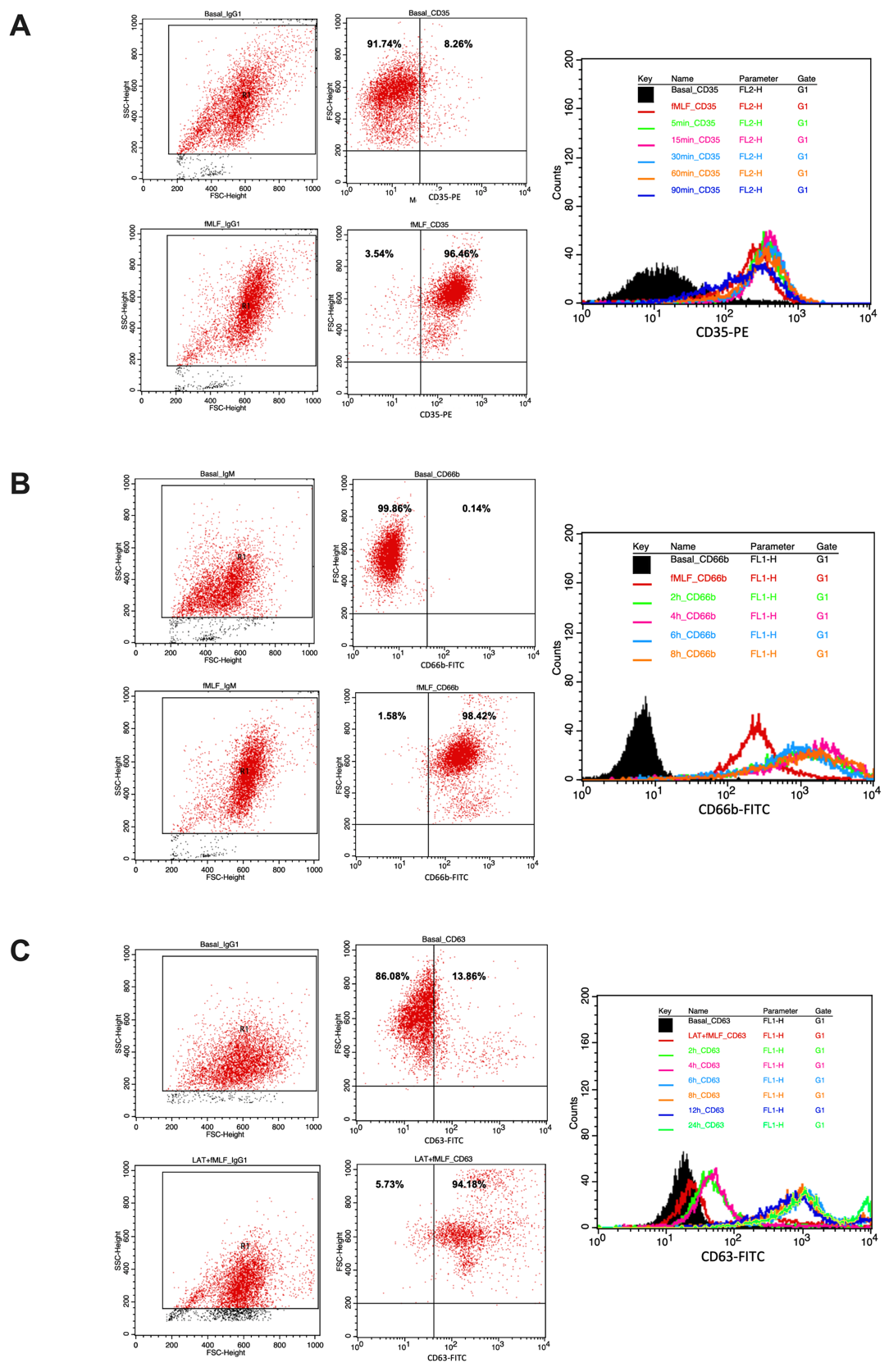
**

**Figure S4. Gating strategy and histograms used in flow cytometric analysis of granule exocytosis by human neutrophils.** Flow cytometry staining was performed using the shown gating strategy and examples of histogram peak shift for each granules marker CD35 (A), CD66b (B) and CD63 (C).

**
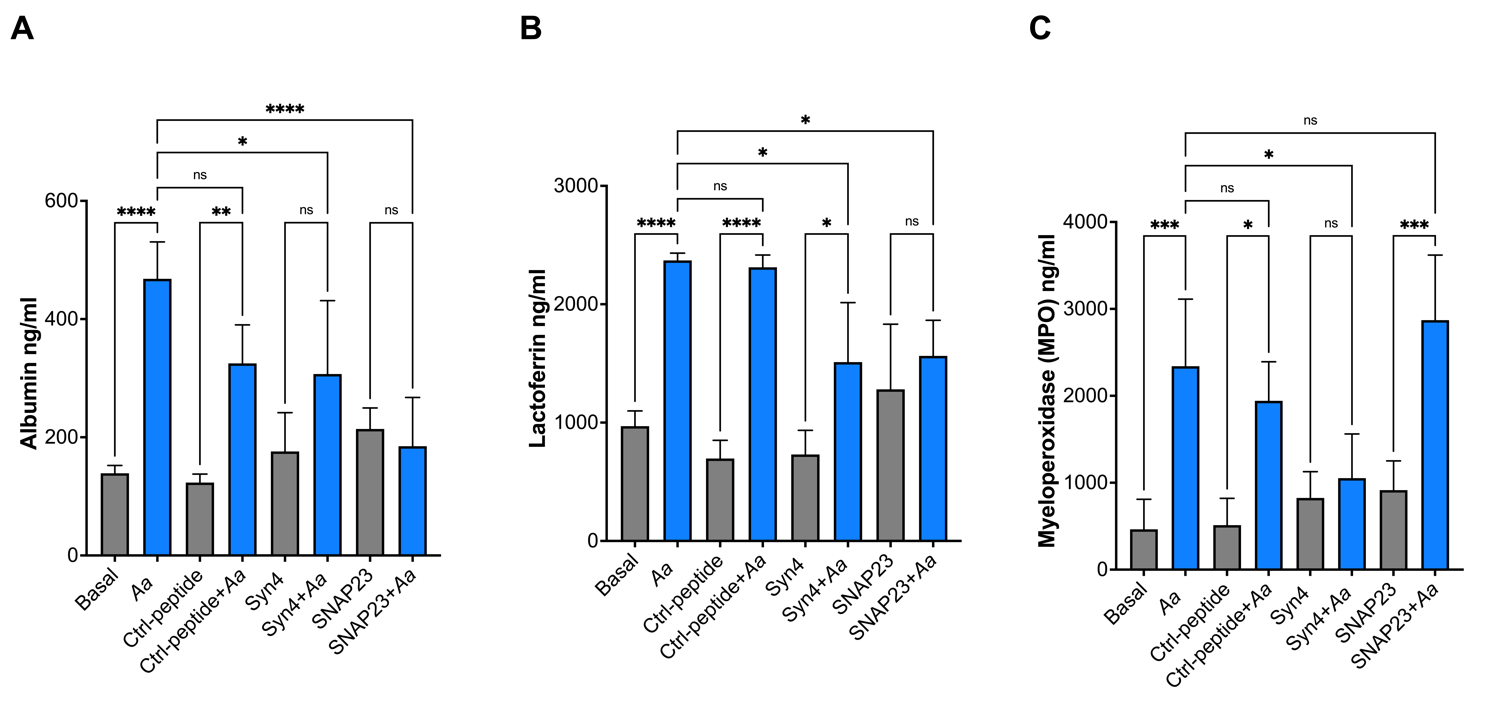
**

**Figure S5. Granule content markers confirm inhibition of granule exocytosis induced by *A. actinomycetemcomitans* in human neutrophils.** Granule fusion inhibition with TAT peptides was confirmed by measuring granule contents. Human neutrophils were pre-treated with Syntaxin 4 (Syn4) or SNAP23 peptide for 15 mins, then challenged with *A. actinomycetemcomitans* (MOI 50) for 15 mins for secretory vesicles **(A)**, for 4h for specific granules **(B)** and for 8h for azurophilic granules **(C)**. Albumin **(A)**, lactoferrin **(B)** and myeloperoxidase **(C)** were measured from collected supernatants and detected by ELISA. The concentration of each content marker is plotted as the mean ± SD of 4 independent experiments. Statistical differences among experimental conditions were analyzed by ordinary one-way ANOVA, followed by Tukey’s post-test. ns: not significant, **p* < 0.05, ***p* < 0.01, ****p* < 0.001, *****p* < 0.0001.

**
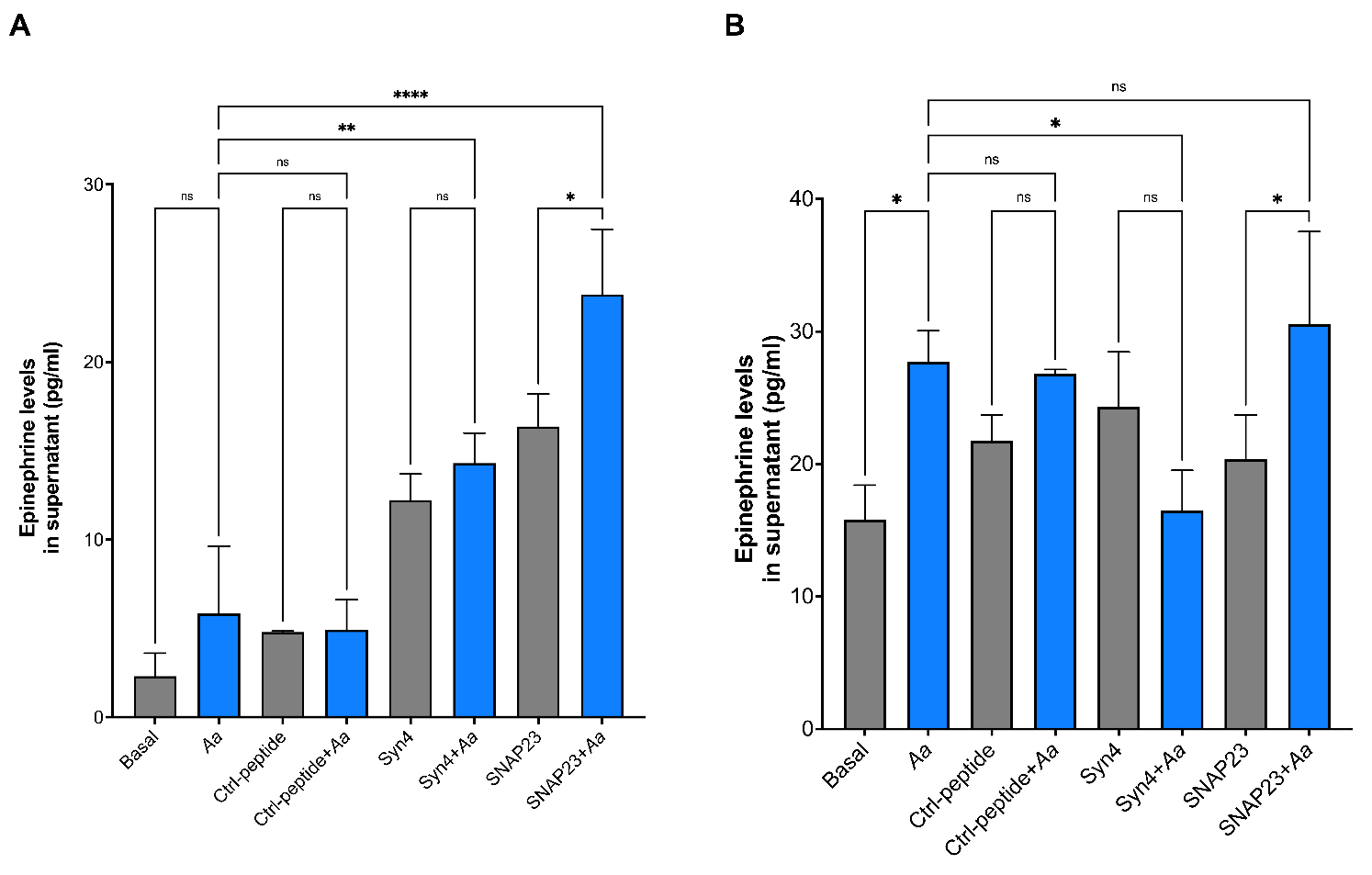
**

**Figure S6. Epinephrine release of TAT peptide treated samples.** Epinephrine storage location was determined by pre-treating human neutrophils with Syntaxin 4 (Syn4) or SNAP23 peptide for 15 mins, then challenged with *A. actinomycetemcomitans* (MOI 50) for 15 mins **(A)** and 2h **(B)**. Epinephrine concentration are plotted as the mean ± SD of 3 independent experiments. Statistical differences among experimental conditions and time points were analyzed by ordinary one-way ANOVA, followed by Tukey’s post-test. ns: not significant, **p* < 0.05, ***p* < 0.01, *****p* < 0.0001.
